# Isolation of a Complete Circular Virus Genome Sequence from an Alaskan Black-Capped Chickadee (*Poecile atricapillus*) Gastrointestinal Tract Sample

**DOI:** 10.1128/genomeA.01081-15

**Published:** 2015-09-24

**Authors:** Zachary R. Hanna, Charles Runckel, Jérôme Fuchs, Joseph L. DeRisi, David P. Mindell, Caroline Van Hemert, Colleen M. Handel, John P. Dumbacher

**Affiliations:** a California Academy of Sciences, San Francisco, California, USA; b Department of Integrative Biology and Museum of Vertebrate Zoology, University of California Berkeley, Berkeley, California, USA; c Department of Biochemistry and Biophysics, University of California San Francisco, San Francisco, California, USA; d Howard Hughes Medical Institute, Bethesda, Maryland, USA; e Alaska Science Center, U.S. Geological Survey, Anchorage, Alaska, USA

## Abstract

We report here the genome sequence of a circular virus isolated from samples of an Alaskan black-capped chickadee (*Poecile atricapillus*) gastrointestinal tract. The genome is 2,152 bp in length and is most similar (30 to 44.5% amino acid identity) to the genome sequences of other single-stranded DNA (ssDNA) circular viruses belonging to the gemycircularvirus group.

## GENOME ANNOUNCEMENT

We isolated a circular viral genome from buccal and cloacal swabs sampled from an Alaskan black-capped chickadee (*Poecile atricapillus*) with avian keratin disorder (1) in Anchorage, AK, on 21 December 2010 and preserved it in RNA*later* (Life Technologies). We extracted DNA and RNA using a ZR viral RNA kit (Zymo Research). We split the extracted nucleic acids into three separate 3-µl aliquots, leaving one aliquot unmodified. We treated the second aliquot with T7 exonuclease and ExoI (New England BioLabs [NEB]) for 1 h at 37°C, and then 20 min at 65°C to eliminate noncircular DNA. We trapped circular DNA present in the third aliquot by adding 50 µl of 0.8% low-melting-point agarose gel melted in Tris-acetate-EDTA buffer, cooling and solidifying the gel, running under a 100-V current for 1 h, and extracting DNA from the gel (2). Next, we performed a multiple displacement amplification (MDA) using Phi29 polymerase (NEB) on each aliquot and precipitated DNA in isopropanol. We pooled equal masses of the three aliquots and then enzymatically cut the DNA with six different 6-cutter restriction enzymes, separated the digested DNA on an agarose gel, and cut out and extracted DNA from the sharpest visualized band, an ~2-kb fragment from the AvrII (NEB) digest. We cloned the gel-extracted DNA, amplified colonies, and sequenced them using an ABI 3130xl.

We assembled sequences and produced subsequent assemblies using the Geneious *de novo* assembler [version 8.1.5; Biomatters (http://www.geneious.com)]. Using the alignment tool BLASTx, we searched the NCBI nonredundant protein sequences collection (3–5), finding matches of our assemblies to circular virus genome capsid and replication gene sequences. We designed primers with reference to the circular virus gene sequences, used them to amplify the remaining circular genomic sequences from the exonuclease and untreated aliquot MDA reaction products, and sequenced them on an ABI 3130xl. We assembled a complete circular genome of length 2,152 bp with two large bidirectionally oriented open reading frames (ORFs). One ORF (protein ID AKU89609) encodes a viral capsid protein and the other (AKU89610) a replicase protein. Using primers designed from the genome, we unsuccessfully attempted to amplify the virus from samples from 17 additional Alaskan *P. atricapillus* birds with avian keratin disorder.

We searched for publicly available sequences most similar to the assembled genome using the alignment tool BLASTx with a word size of 3 to search the NCBI nonredundant protein sequences collection (3–6). Ranked by bit score and discarding hits to hypothetical and unverified bacterial proteins, the top hits were capsid- and replication-associated proteins from viruses in the gemycircularvirus group (7). We performed a MUSCLE (version 3.8.425) (8) alignment of our genome against the 32 gemycircularvirus group complete genomes in NCBI GenBank (3, 6) on 27 May 2015. The alignment resulted in 30 to 44.5% identity with the genomes of the gemycircularvirus group at the amino acid level. Our genome sequence contributes to the resource of circular viral sequences isolated from vertebrate fecal samples (9–11).

### Nucleotide sequence accession numbers.

The genome has been deposited in GenBank under the accession no. KT309029. The version described in this paper is the first version, KT309029.1.
